# Nicotine Content in Swedish-Type Snus Sold in Norway From 2005 to 2020

**DOI:** 10.1093/ntr/ntac006

**Published:** 2022-01-11

**Authors:** Tord Finne Vedøy, Karl Erik Lund

**Affiliations:** Department of Alcohol, Tobacco and Drugs, Norwegian Institute of Public Health, PO Box 222-Skøyen, 0213 Oslo, Norway; Department of Alcohol, Tobacco and Drugs, Norwegian Institute of Public Health, PO Box 222-Skøyen, 0213 Oslo, Norway

## Abstract

**Introduction:**

Use of snus (moist smokeless tobacco) is widespread in Scandinavia and increasingly popular in the U.S. Snus products vary in terms of product design, portion size, and nicotine content. The aim of this study was to examine variations in the nicotine content in snus sold on the Norwegian market from 2005 to 2020.

**Methods:**

We calculated the nicotine content in dry snus in milligram per gram (mg/g) and milligram per serving (mg/s), weighted by the products’ market share from data on nicotine content, water content, and portion size (both for portion and loose snus) for the ten most sold snus products from each of the three largest manufacturers in 2005, 2010, 2015, and 2020.

**Results:**

In all snus products combined, the nicotine content per gram snus (mg/g) increased from 16.3 to 24.1, while nicotine per serving (mg/s) was stable around 13.0. In portion snus, the nicotine content increased for both mg/g and mg/s, most notably from 2005 to 2010. In loose snus, mg/g decreased marginally, while mg/s was stable throughout the period.

**Conclusions:**

In a period with increasing snus use, the nicotine content in snus increased per gram snus, but not per serving. The stability in nicotine per serving is likely due to a decreasing market share of loose snus which accounted for 54% of the snus products in 2005 and 5% in 2020, and which traditionally has a high content of nicotine per serving.

**Implications:**

Use of snus is popular in Scandinavia, most notably in Sweden and Norway, but also increasingly common in Finland, especially among young adults. There are no prior market-based studies of variations in the nicotine content in Swedish snus over time. We found that the average amount of nicotine per gram snus sold on the Norwegian market increased in the period 2005 to 2020, most notably from 2005 to 2010, while the amount of nicotine per serving was stable in the same period, primarily due to a decreasing share of loose snus.

## Introduction

Snus is a smokeless tobacco product traditionally used in Scandinavia, most notably in Sweden and Norway,^1,2^ but also increasingly common in Finland, especially among young adults.^3^ Sales of snus is also steadily increasing in the United States,^4^ and Swedish Match, the largest snus manufacturer in Sweden, has successfully obtained approval from the U.S. Food and Drug Administration (FDA) to market eight snus products as Modified Risk Tobacco Products on the U.S. market.^4,5^ Swedish Match has also sought access to the European market, where sale of snus is currently banned,^6^ and shown interests in Asian and African markets.^7^

Domestic sales of snus in Norway increased from 286 tons per year in 1985 to 1487 in 2019.^8^ These figures, however, do not include snus bought in Sweden, tax free or from other sources that do not provide revenue to the Norwegian state, which has been estimated to make up around 40% of the total consumption in the period 2010–2015.^9^

From 1985 to 2000, the prevalence of snus use was below 5% for men and close to zero for women (16–74 years). After 2000, daily snus use among men rose steadily to 19% in 2019. Among women, the increase began around 10 years later, and in 2019, 7% of women used snus daily. Both the rate of increase and the extent of use has been larger among young adults compared with adult, and daily snus use has been more prevalent compared to daily smoking among young adult men from 2006 and among young adult women from 2011 (16–24 years).^1,10–12^

The increase in snus use has been accompanied by a proliferation of snus products. Traditionally, snus was sold as a standardized tobacco product, in identical paper cans without any flavor descriptor, typically containing 50 g of loose snus, and almost exclusively from one single producer, Swedish Match. After the turn of the millennium, new products were added to the market, new flavors were introduced and cans became increasingly varied with regards to color, shape, size, and weight, most often produced in plastic or tin and typically containing snus in a variety of user-friendly sachets (large, slim, super-slim, mini) made of cellulose. However, the variations of cans with regards to size, color, and font came to an end in 2017, with the introduction of a plain packaging law for tobacco products.

Given the increased variability of the physical properties of snus products, it is likely that the snus also has become more varied in terms of nicotine content. In the case of cigarettes, a study by Connolly et al. published in 2007 demonstrated that both nicotine content (milligram per gram) and nicotine yield (milligram nicotine per cigarette), measured in smoke generated by a smoking machine based on the Massachusetts smoking regimen, increased in major cigarette brands from the late 1997 to 2005.^13^ A later study by Land et al. found increased nicotine yield, but not increased nicotine content in the period 1997 to 2012, using data collected from the annual reports filed with the Massachusetts Department of Public Health by four major manufacturers of cigarettes sold in Massachusetts.^14^

To date, there are no market-based studies of changes in the nicotine content in Swedish snus over time. The aims of this study were to examine (1) the market composition of snus brands and snus products and (2) calculate the nicotine content in snus sold on the Norwegian market, per gram and per serving, in 2005, 2010, 2015, and 2020.

## Methods

In 2018, the Norwegian Department of Health and Care Services commissioned the Norwegian Institute of Public Health to write a report on the health effects of snus.^1^ To assess the nicotine content in snus, we contacted the joint office for the tobacco industry in Norway in October 2018 and requested information on the nicotine content and market shares (originally collected by Nielsen at retail outlets) of the 10 most sold products from each of the three largest snus manufactures in the Norwegian market (British–American Tobacco, Skruf/Imperial, and Swedish Match) in 2005, 2010, and 2015. Section 38 in the Norwegian Tobacco Control Act gives the Directorate of Health the authority to request product information from tobacco producers.^15^

In January 2019, we received information from the three manufacturers and recognized the need for additional information about water content, portion size in grams and net weight of snus cans of each product. We received an updated spreadsheet in April 2019. In April 2021, after a third request, we received additional data on snus sold in 2020. This last spreadsheet also contained information for 2019, making it possible to check if the snus market in 2020 was extraordinary, due to the covid-19 epidemic (Supplementary File 1).

From these data, we calculated the arithmetic mean content of nicotine in milligrams per gram (mg/g) and milligrams per serving (mg/s) in loose snus and portion snus, using market share as an analytic weight in Stata 15. The calculations of mg/g were based on the dry weight of snus to account for variations in water content. In the case of loose snus, we based our calculations of nicotine per serving on an assumed portion weight of 2.5 g, based on the median serving size from a study by Digard et al.^16^

Because the data in principle represent a complete “population,” and not a sample of an underlying distribution, we will not provide inferential statistics. However, we conduct sensitivity analyses to address possible bias resulting from not having data on all products on the market.

## Results

The ten most sold snus products from each of the three largest manufacturers accounted for 94% of the total domestic snus market in 2005, 88% in 2010, 79% in 2015, and 74% in 2020 (Supplementary File 1). The market share of loose snus declined sharply from 54% in 2005 to 26% in 2010 to 10% in 2015 and 5% in 2020.

**Figure 1.**
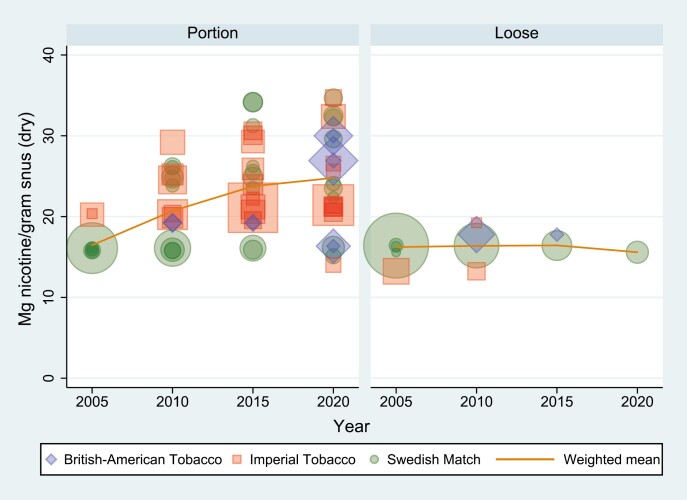
Distribution and weighted mean of milligrams nicotine per gram dry snus (mg/g) 2005, 2010, 2015, and 2020 across product type and manufacturer. Marker shape denotes manufacturer, and marker size denotes market share.

**Figure 2.**
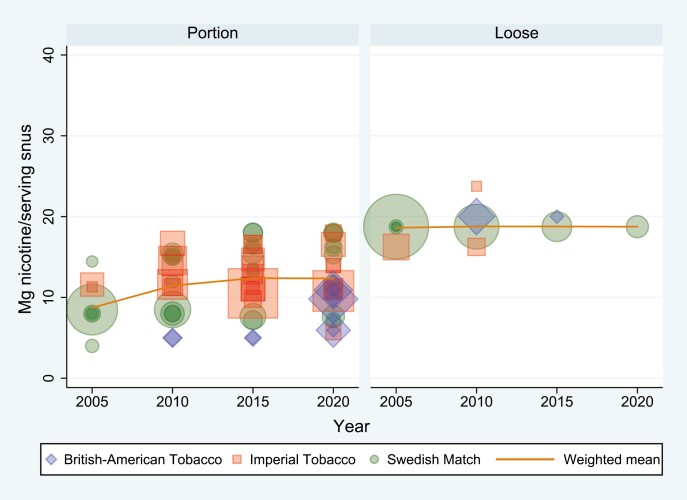
Distribution and weighted mean of milligrams nicotine per serving (mg/s) snus in 2005, 2010, 2015, and 2020 across product type and manufacturer. Marker shape denotes manufacturer, and marker size denotes market share.

Figures 1 and 2 display the weighted means of milligram nicotine per gram dry snus (mg/g) and per serving (mg/s) for portion and loose snus. The size of the markers indicates market share and the color indicates manufacturer. More detailed results (mg/g and mg/s) for portion and loose snus across all years and manufacturers are presented in Supplementary File 2.

The content of nicotine per gram dry snus (mg/g) increased from 16.3 in 2005 to 19.4 in 2010, 22.8 in 2015, and 24.1 in 2020. In the same period, the weighted amount of nicotine per serving (mg/s) remained stable (14.4 in 2005, 13.6 in 2010, 13.2 in 2015, and 12.8 in 2020). However, when considering product type separately (loose or portion), there was an increased content of nicotine in portion snus, both per gram (from 16.5 in 2005, 20.7 in 2010, 23.8 in 2015 to 24.8 in 2020) and per serving (from 8.7 in 2005, 11.5 in 2010, 12.4 in 2015 to 12.3 in 2020). In loose snus, mg/g decreased marginally (from 16.2 in 2005, 16.4 in 2010 and 2015 to 15.6 in 2020), while mg/s was stable (from 18.6 in 2005 to 18.8 in 2010, 2015, and 2020).

## Discussion

According to this study, the content of nicotine per gram dry snus (mg/g) sold on the Norwegian market increased by 47.7% from 2005 to 2020, while the content of nicotine per serving (mg/s) decreased by 11.1%. When decomposing the snus market into portion and loose snus, we found that the nicotine content in portion snus increased, both measured as mg/g and mg/s, and most of the increase in mg/s took place between 2005 and 2010. In loose snus, mg/s remained stable and mg/g decreased marginally. The increase in milligram nicotine per gram snus and new product designs of portion snus is in line with previous reports from the U.S. smokeless tobacco market.^17,18^

The lack of an increase in overall nicotine content per serving (mg/s) is likely the result of the decreasing market share of loose snus, which most often has a high nicotine content per serving due to larger portion size (2.5 g). This has likely offset the increase in mg/s in portion snus, most notably from 2005 to 2010. The larger increase in nicotine measured as mg/g (50.6%) compared with mg/s (41.5%) in portion snus from 2005 to 2020 is likely a result of smaller portions. In our data, the weighted average mass of dry portions decreased by 6.3% from 2005 to 2020.

We do not know if the increase in milligram nicotine per gram snus is user- or industry-driven from this data set. Compared to 2005, snus products in 2020 were more varied in terms of flavor and portion size, and with a generally higher nicotine content (mg/g). A study from the United States found that both the content of free nicotine, number and variety of sub brands and advertising increased from 2000 to 2006—a period with increasing snus use among young people.^18^ The authors argued that these changes were a result of tobacco industry strategies. Given that the tax on snus in Norway increased from 58 to 109 NOK per 100 g (approximately 9 and 12 USD) in the period 2005 to 2020, the increase of nicotine in mg/g could be a strategy to reduce costs by reducing the weight of snus while keeping the mg/s stable. However, it may also be that since snus use has been relatively popular in Norway for several decades, snus users have become older,^1^ more tolerant to nicotine and more interested in specific product characteristics.

### Strengths and Limitations

This is the first market-based study of variations in the nicotine content in Swedish snus over time. To check the potential impact of products not included in the sample (5.9% in 2005 and 25.6% in 2020) on the nicotine content, we calculated two complementary scenarios where the unknown share was identical to products with the highest/lowest nicotine content (both mg/g and mg/s) from the same year (Supplementary File 3). In both scenarios, the estimated mg/g and mg/s were similar to the observed figures.

Some limitations must be addressed. First, data was provided by the snus manufacturers and some of these data could not be verified, such as the water content of snus. However, an analysis of American and Swedish smokeless tobacco sold in the period 2008–2009, and which included some of the products on the Norwegian market in 2010, showed to a large degree a similar nicotine content.^19^

Second, we do not know the type and content of the share of snus products used in Norway, but bought elsewhere. These are estimated to account for 40% of all snus used in Norway.^8^ While snus products sold in duty-free outlets, estimated to comprise around 15% of the total consumption,^8^ are similar to snus products in Norway, snus bought in Sweden may differ, also identical sounding products, due to different national regulations. Nevertheless, it seems likely that Norwegians who travel to Sweden to buy snus, buy snus they are familiar with. One exception may be nicotine pouches that contain nicotine, but not tobacco—a product that has not been allowed on the Norwegian market due to a regulation blocking sales of new nicotine products.^15^

Third, as shown by Alpert et al.,^18^ tobacco manufacturers can control the amount of uniounized (free) nicotine by increasing pH. We do not know if this applies to snus on the Norwegian market, as we did not have data on pH or (free) nicotine. However, if snus manufacturers have manipulated the pH, trends in unionized nicotine may be different from the trends in nicotine content reported here. Future studies should examine this possibility.

Lastly, covid-19-related travel restrictions resulted in a dramatic decrease of cross-border trade and duty-free sales.^20^ To check whether the snus market in 2020 was extraordinary, we compared market shares and product portfolio in 2020 with data from 2019. The weighted nicotine content (both mg/g and mg/s) were almost identical across the 2 years, both for loose snus and for portion snus.

## Supplementary Material

A Contributorship Form detailing each author’s specific involvement with this content, as well as any supplementary data, are available online at https://academic.oup.com/ntr.

ntac006_suppl_Supplementary_Data_S1

ntac006_suppl_Supplementary_Data_S2

ntac006_suppl_Supplementary_Data_S3

ntac006_suppl_Supplementary_Data_S4

## Data Availability

The data underlying this article are available in the article and in its online Supplementary Material.
