# Supplementary material for: Nicotine Content in Swedish-Type Snus Sold in Norway From 2005 to 2020
Source: Nicotine Tob Res. 2022 Jan 11;24(7):1130–3. doi: 10.1093/ntr/ntac006 (PMC9199937; doi:10.1093/ntr/ntac006)
Supplement: ntac006_suppl_Supplementary_Data_S1 [file ntac006_suppl_supplementary_data_s1.docx]

**Supplementary File 1: Market share, weight per can, weight per portion, nicotine concentration and water concentration of the 10 most sold snus products from Swedish Match, Imperial and British-American Tobacco, 2005, 2010, 2015, 2019 and 2020.**

| Year | Product name | Brand | Market share | Grams per can* | Grams per portion* | Percent nicotine* | Percent water* |
| --- | --- | --- | --- | --- | --- | --- | --- |
| 2005 | General Loose | Swedish Match | 47.30 | 50.00 | 2.50 | 0.75 | 54.30 |
| 2005 | General Original Portion | Swedish Match | 27.20 | 24.00 | 1.00 | 0.85 | 47.20 |
| 2005 | Gøteborgs Rapé White Portion | Swedish Match | 2.90 | 24.00 | 1.00 | 0.80 | 49.40 |
| 2005 | Ettan Loose | Swedish Match | 2.10 | 50.00 | 2.50 | 0.75 | 54.40 |
| 2005 | General White Portion | Swedish Match | 1.80 | 24.00 | 1.00 | 0.80 | 49.70 |
| 2005 | General Original Mini Portion | Swedish Match | 1.80 | 10.00 | 0.50 | 0.80 | 48.90 |
| 2005 | General Original Maxi Portion | Swedish Match | 1.30 | 24.00 | 1.70 | 0.85 | 47.50 |
| 2005 | Catch White Portion | Swedish Match | 1.00 | 24.00 | 1.00 | 0.80 | 49.30 |
| 2005 | Grovsnus Loose | Swedish Match | 1.00 | 50.00 | 2.50 | 0.75 | 54.10 |
| 2005 | Gøteborgs Rapé Loose | Swedish Match | 0.80 | 50.00 | 2.50 | 0.75 | 51.80 |
| 2005 | Skruf Original Portion | Imperial | 3.40 | 24.00 | 1.00 | 1.16 | 42.70 |
| 2005 | Skruf Original Loose | Imperial | 2.90 | 50.00 | 2.50 | 0.65 | 50.90 |
| 2005 | Skruf Tranbär Portion | Imperial | 0.60 | 24.00 | 1.00 | 1.13 | 44.50 |
| 2010 | General Loose | Swedish Match | 22.40 | 45.00 | 2.50 | 0.75 | 54.30 |
| 2010 | General Original Portion | Swedish Match | 13.80 | 24.00 | 1.00 | 0.85 | 47.20 |
| 2010 | General White Portion | Swedish Match | 5.70 | 24.00 | 1.00 | 0.80 | 49.70 |
| 2010 | General Extra Strong Original Portion | Swedish Match | 4.80 | 24.00 | 1.10 | 1.40 | 43.70 |
| 2010 | The Lab Strong Original Portion | Swedish Match | 3.00 | 24.00 | 1.00 | 1.50 | 42.80 |
| 2010 | Gøteborgs Rapé White Portion | Swedish Match | 2.80 | 24.00 | 1.00 | 0.80 | 49.40 |
| 2010 | Nick & Johnny Strong Original Portion | Swedish Match | 2.60 | 24.00 | 1.10 | 1.40 | 43.40 |
| 2010 | Catch White Eucalyptus Portion | Swedish Match | 2.60 | 24.00 | 1.00 | 0.80 | 49.30 |
| 2010 | General Strong Long Portion | Swedish Match | 1.90 | 24.00 | 1.00 | 1.50 | 42.50 |
| 2010 | General White Strong Portion | Swedish Match | 1.80 | 24.00 | 1.00 | 1.20 | 49.70 |
| 2010 | Skruf Original Portion | Imperial | 5.59 | 24.00 | 1.00 | 1.16 | 42.70 |
| 2010 | Skruf Strong Portion | Imperial | 4.87 | 24.00 | 1.00 | 1.46 | 40.50 |
| 2010 | Skruf Extra Strong Portion | Imperial | 3.68 | 24.00 | 1.00 | 1.67 | 42.80 |
| 2010 | Skruf Strong White Portion | Imperial | 2.75 | 24.00 | 1.00 | 1.40 | 44.50 |
| 2010 | Skruf Original White Portion | Imperial | 2.59 | 24.00 | 1.00 | 1.15 | 42.10 |
| 2010 | Skruf Tranbär Portion | Imperial | 1.73 | 24.00 | 1.00 | 1.13 | 44.50 |
| 2010 | Skruf Strong Loose | Imperial | 1.30 | 45.00 | 2.50 | 0.65 | 50.90 |
| 2010 | Skruf Extra Strong Loose | Imperial | 0.42 | 45.00 | 2.50 | 0.95 | 50.60 |
| 2010 | Blå Snus Loose | British-American Tobacco | 1.50 | 42.00 | 2.50 | 0.80 | 55.00 |
| 2010 | Mocca Pink Portion | British-American Tobacco | 0.91 | 8.00 | 0.40 | 1.25 | 35.00 |
| 2010 | Mocca Black Portion | British-American Tobacco | 0.79 | 8.00 | 0.40 | 1.25 | 35.00 |
| 2015 | General Classic Loose | Swedish Match | 9.88 | 42.00 | 2.50 | 0.75 | 54.30 |
| 2015 | General Classic Original Portion | Swedish Match | 6.96 | 21.60 | 0.90 | 0.85 | 47.20 |
| 2015 | General Classic Extra Strong Portion | Swedish Match | 4.18 | 24.00 | 1.10 | 1.40 | 43.70 |
| 2015 | General G.3 Extra Strong Portion | Swedish Match | 3.84 | 21.60 | 0.90 | 2.00 | 41.50 |
| 2015 | The Lab Extra Strong Portion | Swedish Match | 3.84 | 21.60 | 0.90 | 2.00 | 41.40 |
| 2015 | General Classic White Portion | Swedish Match | 3.72 | 21.60 | 0.90 | 0.80 | 49.70 |
| 2015 | Nick & Johnny Crushed Ice Extra Strong Portion | Swedish Match | 2.36 | 24.00 | 1.10 | 1.50 | 41.20 |
| 2015 | General G.3 Extra Strong White Portion | Swedish Match | 2.01 | 21.60 | 0.90 | 1.80 | 42.40 |
| 2015 | Nick & Johnny Crushed Ice Extra Strong White Portion | Swedish Match | 1.81 | 22.00 | 1.10 | 1.20 | 49.00 |
| 2015 | The LaB Strong Portion | Swedish Match | 1.57 | 21.60 | 0.90 | 1.50 | 42.80 |
| 2015 | Skruf Fresh White Slim Portion | Imperial | 15.43 | 16.80 | 0.70 | 1.50 | 28.90 |
| 2015 | Skruf Original White Portion | Imperial | 3.54 | 22.00 | 0.92 | 1.20 | 43.40 |
| 2015 | Skruf Original Portion | Imperial | 3.42 | 22.00 | 0.92 | 1.20 | 41.70 |
| 2015 | Skruf Fresh Extra Strong White Slim Portion | Imperial | 3.19 | 16.80 | 0.70 | 2.10 | 28.30 |
| 2015 | Skruf Strong White Portion | Imperial | 2.88 | 22.00 | 0.92 | 1.50 | 42.10 |
| 2015 | Skruf Strong Portion | Imperial | 2.37 | 22.00 | 0.92 | 1.40 | 41.60 |
| 2015 | Skruf Extra Strong Portion | Imperial | 2.11 | 22.00 | 0.92 | 1.80 | 40.10 |
| 2015 | Skruf Extra Strong White Portion | Imperial | 1.96 | 22.00 | 0.92 | 1.80 | 41.20 |
| 2015 | Skruf Nordic White Licorice Slim Portion | Imperial | 1.75 | 16.80 | 0.70 | 1.40 | 28.40 |
| 2015 | Skruf Original White Slim Portion | Imperial | 1.01 | 16.80 | 0.70 | 1.60 | 28.00 |
| 2015 | Mocca Pink Portion | British-American Tobacco | 0.73 | 8.00 | 0.40 | 1.25 | 35.00 |
| 2015 | Mocca Black Portion | British-American Tobacco | 0.55 | 8.00 | 0.40 | 1.25 | 35.00 |
| 2015 | Blå Snus Loose | British-American Tobacco | 0.21 | 42.00 | 2.50 | 0.80 | 55.00 |
| 2019 | General Classic Loose | Swedish Match | 5.51 | 42.00 | 2.50 | 0.75 | 51.90 |
| 2019 | General Original Portion | Swedish Match | 5.20 | 21.60 | 0.90 | 0.85 | 47.50 |
| 2019 | General G.3 Extra Strong Portion | Swedish Match | 4.30 | 21.60 | 0.90 | 2.00 | 38.40 |
| 2019 | The Lab Extra Strong Portion | Swedish Match | 3.52 | 21.60 | 0.90 | 2.00 | 38.00 |
| 2019 | General G.3 Extra Strong White Portion | Swedish Match | 3.13 | 21.60 | 0.90 | 1.80 | 39.20 |
| 2019 | General Extra Strong Original Portion | Swedish Match | 3.11 | 24.00 | 1.09 | 1.40 | 40.50 |
| 2019 | General G.3 Volt Super Strong Portion | Swedish Match | 3.02 | 16.60 | 0.69 | 2.60 | 25.00 |
| 2019 | General White Portion | Swedish Match | 2.33 | 21.60 | 0.90 | 0.80 | 47.00 |
| 2019 | Nick & Johnny Crushed Ice Extra Strong | Swedish Match | 2.21 | 24.00 | 1.09 | 1.50 | 37.70 |
| 2019 | Nick & Johnny Crushed Ice Extra Strong White Portion | Swedish Match | 1.41 | 22.00 | 1.00 | 1.20 | 46.40 |
| 2019 | Skruf Fresh White Slim Portion | Imperial | 11.82 | 16.60 | 0.72 | 1.50 | 30.00 |
| 2019 | Skruf Fresh Extra Strong White Slim Portion | Imperial | 4.32 | 16.60 | 0.72 | 2.30 | 29.00 |
| 2019 | Skruf Original White Portion | Imperial | 2.53 | 21.60 | 0.93 | 1.20 | 43.50 |
| 2019 | Skruf Original Portion | Imperial | 2.39 | 21.60 | 0.93 | 1.20 | 41.50 |
| 2019 | Skruf Slim Original White Portion | Imperial | 2.12 | 16.60 | 0.72 | 1.50 | 29.00 |
| 2019 | Skruf Slim Nordic White Portion | Imperial | 1.86 | 16.60 | 0.72 | 1.50 | 30.00 |
| 2019 | Skruf Strong White Portion | Imperial | 1.58 | 21.60 | 0.93 | 1.50 | 43.50 |
| 2019 | Skruf Slim Fresh Ultra Strong White Portion | Imperial | 1.55 | 16.60 | 0.72 | 2.50 | 28.00 |
| 2019 | Skruf Strong Portion | Imperial | 1.37 | 21.60 | 0.93 | 1.50 | 41.50 |
| 2019 | Skruf Extra Strong White Portion | Imperial | 1.18 | 16.60 | 0.93 | 1.80 | 43.50 |
| 2019 | Epok Ice Blue Intense Portion | British-American Tobacco | 5.09 | 16.80 | 0.70 | 1.40 | 48.00 |
| 2019 | Epok Arctic Blue Portion | British-American Tobacco | 2.47 | 16.80 | 0.70 | 0.85 | 48.00 |
| 2019 | Epok Freeze Extra Strong Portion | British-American Tobacco | 1.74 | 16.80 | 0.70 | 1.56 | 48.00 |
| 2019 | Epok Zest Green Intense Portion | British-American Tobacco | 0.59 | 16.80 | 0.70 | 1.40 | 48.00 |
| 2020 | General Loose | Swedish Match | 5.40 | 42.00 | 2.50 | 0.75 | 51.90 |
| 2020 | General Original Portion | Swedish Match | 5.12 | 21.60 | 0.90 | 0.85 | 47.50 |
| 2020 | General G.3 Extra Strong Portion | Swedish Match | 3.86 | 21.60 | 0.90 | 2.00 | 38.40 |
| 2020 | General G.3 Volt Super Strong Portion | Swedish Match | 3.47 | 16.60 | 0.69 | 2.60 | 25.00 |
| 2020 | General Extra Strong Original Portion | Swedish Match | 3.33 | 24.00 | 1.09 | 1.40 | 40.50 |
| 2020 | General G.3 Extra Strong Slim White Portion | Swedish Match | 3.19 | 21.60 | 0.90 | 1.80 | 39.20 |
| 2020 | The Lab Extra Strong Portion | Swedish Match | 3.01 | 21.60 | 0.90 | 2.00 | 38.00 |
| 2020 | General White Portion | Swedish Match | 2.34 | 21.60 | 0.90 | 0.80 | 47.00 |
| 2020 | Nick & Johnny Crushed Ice Extra Strong Portion | Swedish Match | 2.09 | 24.00 | 1.09 | 1.50 | 37.70 |
| 2020 | Nick & Johnny Crushed Ice Extra Strong White Portion | Swedish Match | 1.31 | 22.00 | 1.00 | 1.20 | 46.40 |
| 2020 | Skruf Slim Fresh White Portion | Imperial | 10.48 | 16.60 | 0.72 | 1.50 | 30.00 |
| 2020 | Skruf Slim Fresh Extra Strong White Portion | Imperial | 3.57 | 16.60 | 0.72 | 2.30 | 29.00 |
| 2020 | Skruf Original White Portion | Imperial | 2.22 | 21.60 | 0.93 | 1.20 | 43.50 |
| 2020 | Skruf Original Portion | Imperial | 1.98 | 21.60 | 0.93 | 1.20 | 41.50 |
| 2020 | Skruf Slim White Portion | Imperial | 1.96 | 16.60 | 0.72 | 1.50 | 29.00 |
| 2020 | Skruf Slim Nordic White Portion | Imperial | 1.76 | 16.60 | 0.72 | 1.50 | 30.00 |
| 2020 | Skruf Slim Fresh Ultra Strong White Portion | Imperial | 1.55 | 16.60 | 0.72 | 2.50 | 28.00 |
| 2020 | Skruf Super White Fresh | Imperial | 1.40 | 17.30 | 0.71 | 0.80 | 43.00 |
| 2020 | Skruf Strong White Portion | Imperial | 1.34 | 21.60 | 0.93 | 1.50 | 43.50 |
| 2020 | Skruf Strong Portion | Imperial | 1.15 | 21.60 | 0.93 | 1.50 | 41.50 |
| 2020 | Epok Ice Blue Intense Portion | British-American Tobacco | 5.86 | 16.80 | 0.70 | 1.40 | 48.00 |
| 2020 | Epok Freeze Extra Strong Portion | British-American Tobacco | 3.62 | 16.80 | 0.70 | 1.56 | 48.00 |
| 2020 | Epok Arctic Blue Portion | British-American Tobacco | 2.85 | 16.80 | 0.70 | 0.85 | 48.00 |
| 2020 | Epok Zest Green Intense Portion | British-American Tobacco | 0.57 | 16.80 | 0.70 | 1.40 | 48.00 |
| 2020 | Epok Winter Chill Extra Strong Portion | British-American Tobacco | 0.51 | 16.80 | 0.70 | 1.56 | 48.00 |
| 2020 | Epok Tropic Breeze Portion | British-American Tobacco | 0.45 | 16.80 | 0.70 | 0.85 | 48.00 |

*Measured as is/in moist product
