# Supplementary material for: Nicotine Content in Swedish-Type Snus Sold in Norway From 2005 to 2020
Source: Nicotine Tob Res. 2022 Jan 11;24(7):1130–3. doi: 10.1093/ntr/ntac006 (PMC9199937; doi:10.1093/ntr/ntac006)
Supplement: ntac006_suppl_Supplementary_Data_S2 [file ntac006_suppl_supplementary_data_s2.docx]

**Supplementary File 2**

**Weighted means (numbers of products, n) of milligrams nicotine per gram dry snus (mg/g) in 2005, 2010, 2015, 2019 and 2020**

|  | **Milligrams per gram** | | | | | |
| --- | --- | --- | --- | --- | --- | --- |
| **Year** | **Total** |  |  |  |  |  |
| **2005** | 16.3 (13) |  |  |  |  |  |
| **2010** | 19.4 (21) |  |  |  |  |  |
| **2015** | 22.8 (23) |  |  |  |  |  |
| **2019** | 24.3 (24) |  |  |  |  |  |
| **2020** | 24.1 (26) |  |  |  |  |  |
|  | **Product type** | |  |  |  |  |
| **Year** | ***Portion*** | ***Loose*** |  |  |  |  |
| **2005** | 16.5 (8) | 16.2 (5) |  |  |  |  |
| **2010** | 20.7 (17) | 16.4 (4) |  |  |  |  |
| **2015** | 23.8 (21) | 16.4 (2) |  |  |  |  |
| **2019** | 25.0 (23) | 15.6 (1) |  |  |  |  |
| **2020** | 24.8 (25) | 15.6 (1) |  |  |  |  |
|  | **Manufacturer and product type** | | | | | |
|  | ***British-American Tobacco*** | | ***Imperial*** | | ***Swedish Match*** | |
| **Year** | ***Portion*** | ***Loose*** | ***Portion*** | ***Loose*** | ***Portion*** | ***Loose*** |
| **2005** | - | - | 20.3 (2) | 13.2 (1) | 16.0 (6) | 16.4 (4) |
| **2010** | 19.2 (2) | 17.8 (1) | 23.4 (6) | 14.7 (2) | 19.3 (9) | 16.4 (1) |
| **2015** | 19.2 (2) | 17.8 (1) | 23.3 (10) | - | 24.6 (9) | 16.4 (1) |
| **2019** | 24.8 (4) | - | 24.4 (10) | - | 25.8 (9) | 15.6 (1) |
| **2020** | 25.3 (6) | - | 23.6 (10) | - | 25.7 (9) | 15.6 (1) |

**Weighted means (numbers of products, n) of milligrams nicotine per serving snus (mg/s) in 2005, 2010, 2015, 2019 and 2020**

|  | **Milligrams per serving** | | | | | | | | | |
| --- | --- | --- | --- | --- | --- | --- | --- | --- | --- | --- |
| **Year** | **Total** |  |  | |  | |  | |  |  |
| **2005** | 14.4 (13) |  |  | |  | |  | |  |  |
| **2010** | 13.6 (21) |  |  | |  | |  | |  |  |
| **2015** | 13.2 (23) |  |  | |  | |  | |  |  |
| **2019** | 13.2 (24) |  |  | |  | |  | |  |  |
| **2020** | 12.8 (26) |  |  | |  | |  | |  |  |
|  | **Product type** | | |  | |  | |  | |  |
| **Year** | ***Portion*** | ***Loose*** |  | |  | |  | |  |  |
| **2005** | 8.7 (8) | 18.6 (5) |  | |  | |  | |  |  |
| **2010** | 11.5 (17) | 18.8 (4) |  | |  | |  | |  |  |
| **2015** | 12.4 (21) | 18.8 (2) |  | |  | |  | |  |  |
| **2019** | 12.8 (23) | 18.8 (1) |  | |  | |  | |  |  |
| **2020** | 12.3 (25) | 18.8 (1) |  | |  | |  | |  |  |
|  | **Manufacturer and product type** | | | | | | | | |  |
|  | ***British-American Tobacco*** | | ***Imperial*** | | | | ***Swedish Match*** | | |  |
| **Year** | ***Portion*** | ***Loose*** | ***Portion*** | | ***Loose*** | | ***Portion*** | | ***Loose*** |  |
| **2005** | - | - | 11.6 (2) | | 16.3 (1) | | 8.4 (6) | | 18.8 (4) |  |
| **2010** | 5.0 (2) | 20.0 (1) | 13.4 (6) | | 18.1 (2) | | 10.6 (9) | | 18.8 (1) |  |
| **2015** | 5.0 (2) | 20.0 (1) | 12.0 (10) | | - | | 13.2 (9) | | 18.8 (1) |  |
| **2019** | 9.0 (4) | - | 12.6 (10) | |  | | 14.3 (9) | | 18.8 (1) |  |
| **2020** | 9.2 (6) | - | 12.0 (10) | | - | | 14.2 (9) | | 18.8 (1) |  |
