# Supplementary material for: Nicotine Content in Swedish-Type Snus Sold in Norway From 2005 to 2020
Source: Nicotine Tob Res. 2022 Jan 11;24(7):1130–3. doi: 10.1093/ntr/ntac006 (PMC9199937; doi:10.1093/ntr/ntac006)

**Supplementary File 3**

**Nicotine content in snus (milligrams per grams) on the Norwegian market if snus products not included in the data set (Missing) had a nicotine content identical to the snus products with the highest and lowest nicotine content (in milligrams per grams)**


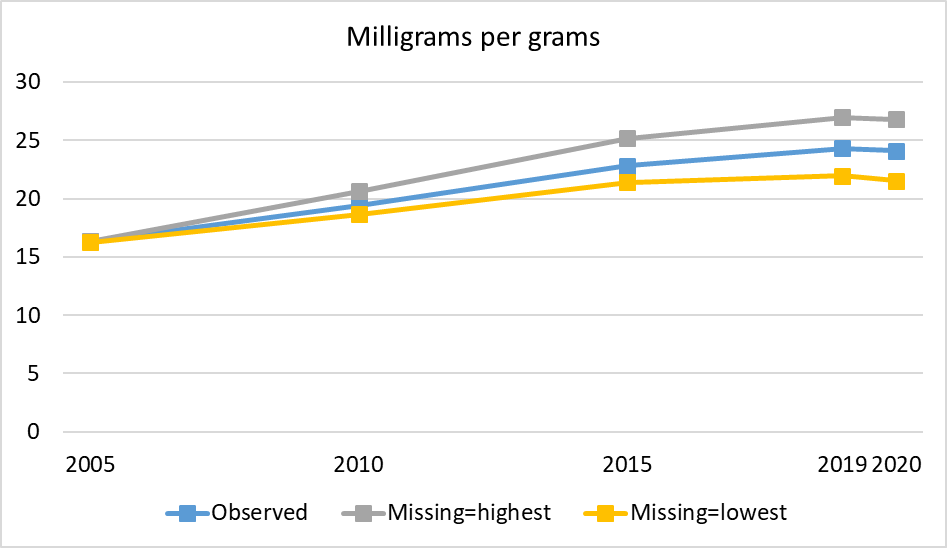


**Nicotine content in snus (milligrams per serving) on the Norwegian market if snus products not included in the data set (Missing) had a nicotine content identical to the snus products with the highest and lowest nicotine content (in milligrams per serving)**


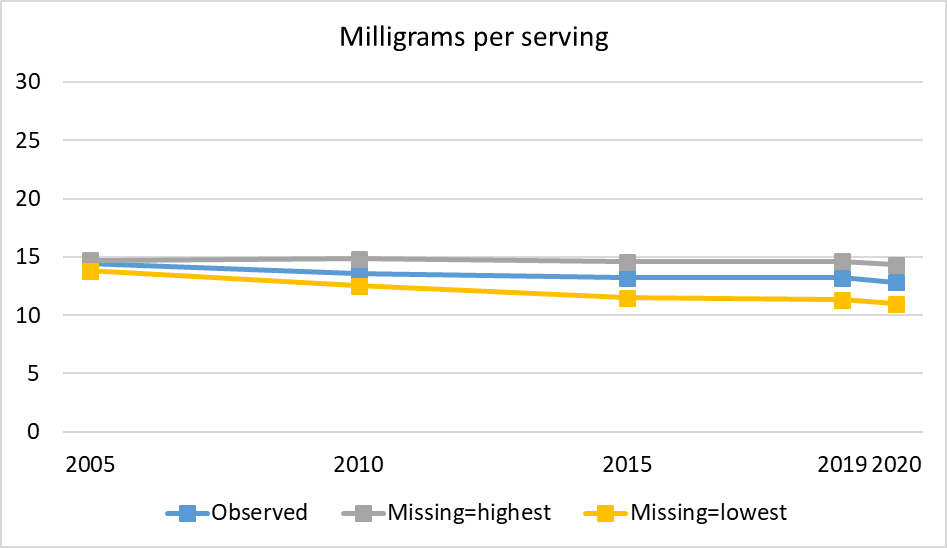

Supplement: ntac006_suppl_Supplementary_Data_S3 [file ntac006_suppl_supplementary_data_s3.docx]
